# Association between the prognostic nutritional index and prognosis in patients with non-small cell lung cancer undergoing curative lung resection: a systematic review and meta-analysis

**DOI:** 10.3389/fonc.2026.1834210

**Published:** 2026-05-21

**Authors:** Shengjie Wu, Xiaojin Jiang, Gang Han

**Affiliations:** Department of Pharmacy, Sir Run Run Shaw Hospital, School of Medicine, Zhejiang University, Hangzhou, China

**Keywords:** meta-analysis, non-small cell lung cancer, PNI, prognostic nutritional index, surgery

## Abstract

**Objective:**

To explore the prognostic value of the prognostic nutritional index (PNI) in patients undergoing curative surgery for non-small cell lung cancer (NSCLC) through a systematic review and meta-analysis.

**Methods:**

Relevant studies published up to October 2025 were searched in PubMed, Embase, Web of Science, and Cochrane. Observational studies assessing the association between PNI and overall, recurrence-free, or cancer-specific survival after curative resection of NSCLC were included. Pooled hazard ratios (HRs) with 95% confidence intervals (CIs) were calculated using a random-effects model. Heterogeneity was evaluated by the I² statistic, with sensitivity analyses and assessment for publication bias.

**Results:**

A total of 11 studies were included in the analysis. Meta-analysis indicated that higher PNI significantly associated with longer OS (HR = 0.79, 95% CI: 0.72–0.87, P<0.00001), RFS (HR = 0.64, 95% CI: 0.51–0.80, P<0.0001), and CSS (HR = 0.50, 95% CI: 0.31–0.80, P = 0.004). Substantial heterogeneity was observed in the OS analysis (I²=88%), but the results were robust in sensitivity analyses. Heterogeneity was low in RFS and CSS (I²=34% and 0%). The association with OS did not change significantly after adjustment for publication bias.

**Conclusion:**

PNI is an independent prognostic indicator for patients undergoing curative surgery for NSCLC. It provides an economical and convenient tool for risk stratification by integrating nutritional and immune function. It is recommended that PNI be incorporated into the preoperative assessment to guide personalized interventions. Future prospective studies are needed to optimize the PNI cutoff value and explore its synergistic effects with multidisciplinary treatments.

**Systematic Review Registration:**

https://www.crd.york.ac.uk/PROSPERO/view/CRD420251237417, identifier CRD420251237417.

## 1 Introduction

Lung cancer is one of the most prevalent and deadly malignancies worldwide, with non-small cell lung cancer (NSCLC) representing the majority of all lung cancer cases (1). In China, the burden of lung cancer is particularly severe (2). According to the latest statistics, lung cancer ranks first in both morbidity and mortality among all cancers, placing significant health and economic pressures on society and individuals alike (3). NSCLC has a variety of treatment options, including surgical resection, chemotherapy, radiotherapy, and the recently emerging immunotherapy (4). For patients with early-stage and locally advanced NSCLC, surgical resection remains the mainstay of curative treatment, particularly curative resection, such as lobectomy or sublobar resection, which significantly improves long-term survival (5). However, even after undergoing radical surgery, some patients remain at risk of recurrence and metastasis, resulting in a highly variable prognosis (6). Therefore, the search for reliable and cost-effective prognostic biomarkers to identify high-risk patients and guide personalized treatment has become a major focus in clinical research. The development and application of biomarkers have long been a key focus in cancer prognostic research (7). While traditional prognostic indicators such as the TNM staging system hold significant value, they fail to fully capture patient heterogeneity, particularly the impact of nutritional and immune status (8).

The Prognostic Nutritional Index (PNI), a simple indicator combining serum albumin and lymphocyte count, was initially developed to evaluate nutritional status and risk in surgical patients (9). In recent years, it has demonstrated significant value in predicting prognosis across various cancers (10). The PNI not only reflects a patient’s nutritional status but is also closely associated with immune function, potentially influencing disease progression by influencing inflammatory responses and anti-tumor immunity (11, 12). A meta-analysis by Wang et al. showed that PNI was significantly associated with the survival of lung cancer patients treated with ICIs and emphasized the predictive role of PNI in immunotherapy (13). However, this study focused primarily on advanced patients and immunotherapy, with limited attention to surgical patients. Surgical resection, as a radical treatment for NSCLC, represents a distinct patient population, and dynamic changes in PNI may more directly reflect postoperative recovery and long-term outcomes (14, 15). In addition, the prognosis of surgical patients is influenced by multiple factors, including age, comorbidities, and surgical approach (16). Whether PNI can provide incremental prognostic information in this context remains to be elucidated.

The purpose of this review was to investigate, through a meta-analysis, the relationship between PNI and long-term survival in patients undergoing curative surgery for NSCLC. Primary outcomes included overall survival (OS), recurrence-free survival (RFS), and cancer-specific survival (CSS). Building on the work of Wang et al., we further highlight the versatility of PNI across different treatment modalities, emphasizing that surgical patients may have unique biological behaviors, such as postoperative inflammatory responses and altered nutritional requirements, which may enhance the prognostic value of PNI. The results of this study aim to provide clinicians with practical tools to help identify high-risk patients after surgery and guide nutritional intervention and follow-up strategies, ultimately improving patient outcomes.

## 2 Methods

### 2.1 Literature search

This meta-analysis was conducted in accordance with the PRISMA 2020 guidelines (17) and was registered in PROSPERO (CRD420251237417). A comprehensive literature search was performed in PubMed, Embase, Web of Science, and Cochrane databases was conducted up to October 2025 to identify studies evaluating the prognostic value of PNI in NSCLC undergoing curative lung resection. The search terms used were: “prognostic nutritional index”, “PNI”, “Non-Small-Cell Lung Carcinoma”. To ensure thoroughness, the reference lists of all included studies were manually screened for further relevant literature. Article selection and evaluation were independently performed by two researchers. Any discrepancies in literature identification were addressed through discussion. Detailed information regarding the search process is available in **Supplementary Table 1**.

### 2.2 Inclusion and exclusion criteria

Inclusion criteria:

P: Patients diagnosed with NSCLC.

E: High PNI.

C: Low PNI.

O: Any survival outcome, including overall survival (OS), relapse-free survival (RFS), cancer-specific survival (CSS) etc.

S: Study design was cohort or case-control study.

Exclusion criteria included study protocols, unpublished research, and non-original publications such as abstracts, letters, comments, corrections, and replies. In addition, single-arm studies and those lacking sufficient data—specifically, studies without sufficient data (the hazard ratio (HR) and the corresponding 95% confidence intervals (CIs) for survival outcomes cannot be directly extracted or calculated from the available data), and reviews were excluded.

### 2.3 Data abstraction

Two investigators independently extracted data, and any discrepancies were resolved by the third author. The following information was extracted from the included studies: name of the first author, year of publication, geographic location, study design, population characteristics, sample size, patient age and sex, PNI cut-off value, and survival outcomes (OS, RFS, CSS). When necessary, corresponding authors were contacted to retrieve missing or incomplete information.

### 2.4 Quality evaluation

The methodological quality of the included cohort and case-control studies was evaluated using the Newcastle-Ottawa Scale (NOS) (18), with scores ranging from 7 to 9 considered indicative of high quality (19). Two reviewers independently conducted the quality assessments, and any disagreements were resolved through discussion.

### 2.5 Statistical analysis

All statistical analyses were performed using Review Manager software (version 5.4.1). Hazard ratios (HRs) were used to synthesize survival outcomes, and each estimate was reported with a 95% confidence interval (CI) based on a random-effects model. Heterogeneity across studies was assessed using Cochran’s Q test (χ²) and the I² statistic (20, 21). Substantial heterogeneity was considered present if the P-value was below 0.1 or the I² value exceeded 50%. For results with >2 included studies, a sensitivity analysis was conducted to assess each included study’s influence on the total metric. Publication bias was evaluated using funnel plot analysis and Egger’s regression test (20) in Stata version 15.1 (StataCorp, College Station, TX, USA), applied to outcomes that included ten or more studies. A P-value less than 0.05 was considered indicative of statistically significant publication bias. For results with publication bias, the trimming and filling method was used to evaluate the impact of publication bias on the results.

## 3 Results

### 3.1 Literature retrieval, study characteristics, and baseline

**Figure 1:**
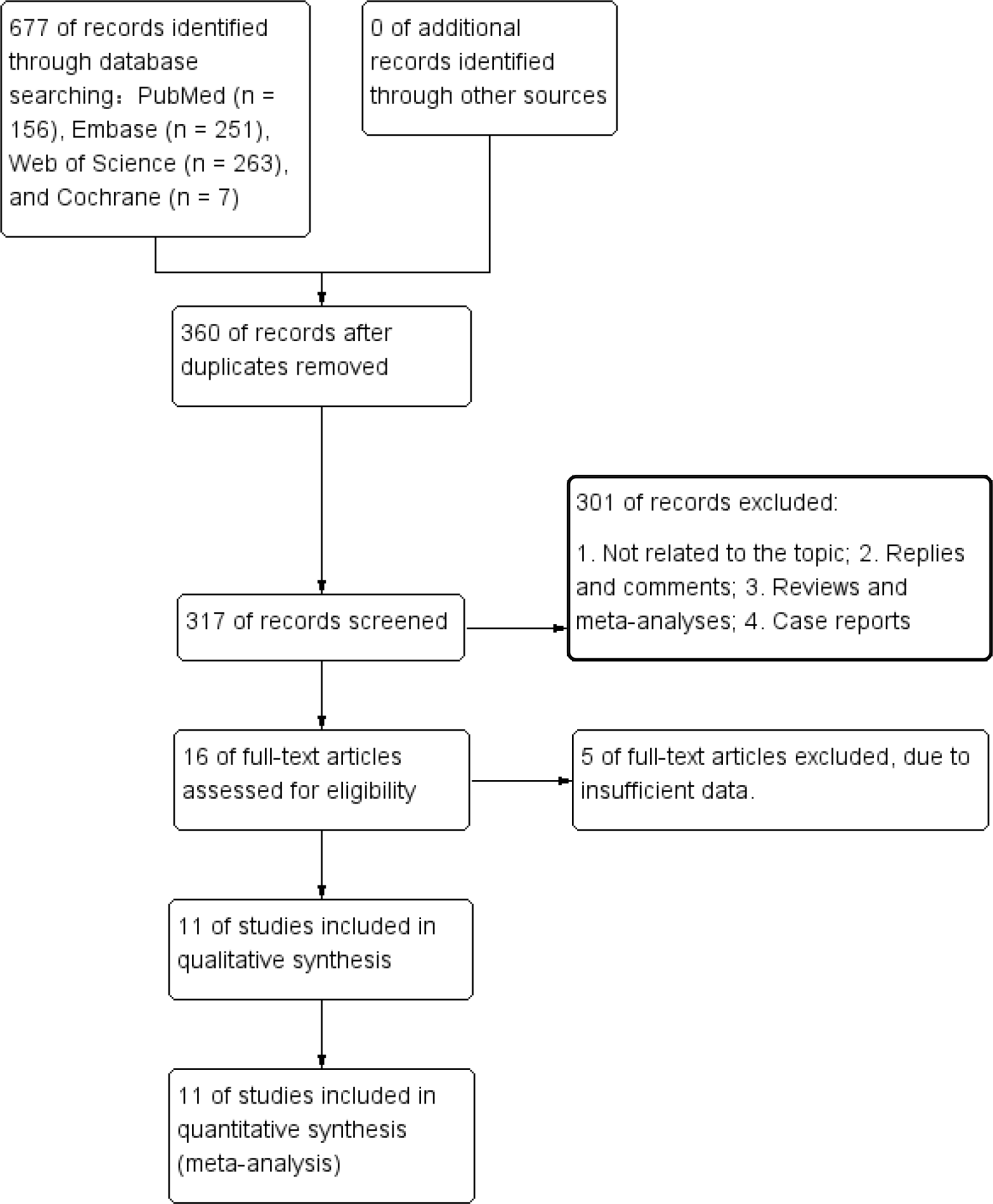
Flowchart of the systematic search and selection process.

The process of study identification and selection is illustrated in the flow diagram (**Figure 1**). A systematic search retrieved 677 records from PubMed (n = 156), Embase (n = 251), Web of Science (n = 263), and the Cochrane Library (n = 7). After the removal of duplicate entries, XXX titles and abstracts were screened for relevance. Ultimately, 11 studies met the inclusion criteria and were incorporated into the meta-analysis (11, 12, 14, 15, 21–27). The key characteristics and quality assessments of the included studies are summarized in **Table 1**. The included literature was published between 2018 and 2025. The PNI cutoff values of the included studies ranged from 39 to 53.95, and the vast majority (10/11) of the studies were from Asia.

**Table 1:** Characteristics and quality assessment of included studies.

Author	Region	Study design	Population	No. of patients	Gender		Age	PNI cut-off	NOS
					Male	Female			
An 2022	Korea	Retrospective cohort	Patients with non-small cell lung cancer undergoing surgical resection	263	170	93	NA	49.6	7
Chen 2018	China	Retrospective cohort	Patients undergoing surgery with stage iB non-small-cell lung cancer	577	410	167	NA	49.55	7
Hayasaka 2024	Japan	Retrospective cohort	Patients with surgically resected non-small cell lung cancer	350	190	160	68	NA	8
Matsubara 2021	Japan	Retrospective cohort	Non-small-cell Lung Cancer Patients Receiving Adjuvant Platinum-based Chemotherapy	110	66	44	64	46.81	7
Okada 2019	Japan	Retrospective cohort	Patients with non-small cell lung cancer (NSCLC) who had undergone major lung resection (lobectomy, bilobectomy, and pneumonectomy) with lymph node dissection	309	188	121	67	48.3	8
Ryu 2023	Korea	Retrospective cohort	Stage I–III NSCLC patients who received postoperative radiotherapy	97	42	55	NA	50.3	8
Sato 2023	Japan	Retrospective cohort	Patients with early-stage non-small cell lung cancer	386	221	165	NA	NA	8
Takahashi 2025	Japan	Retrospective cohort	Patients with resectable non-small cell lung cancer	2770	1674	1096	69	45	7
Taylor 2024	UK	Retrospective cohort	Patients who underwent lung resection (including sublobar wedge resection, anatomical segmentectomy, lobectomy, bilobectomy, or pneumonectomy) for primary NSCLC	5029	2444	2585	68.6	39	7
Tomita 2018	Japan	Retrospective cohort	Patients undergoing Curative Resection for Non-small Cell Lung Cancer	341	173	168	NA	45	7
Zhou 2022	China	Retrospective cohort	Patients with NSCLC who underwent curative resection	64	34	30	63	53.95	7

### 3.2 Prognostic value of PNI for OS

**Figure 2:**
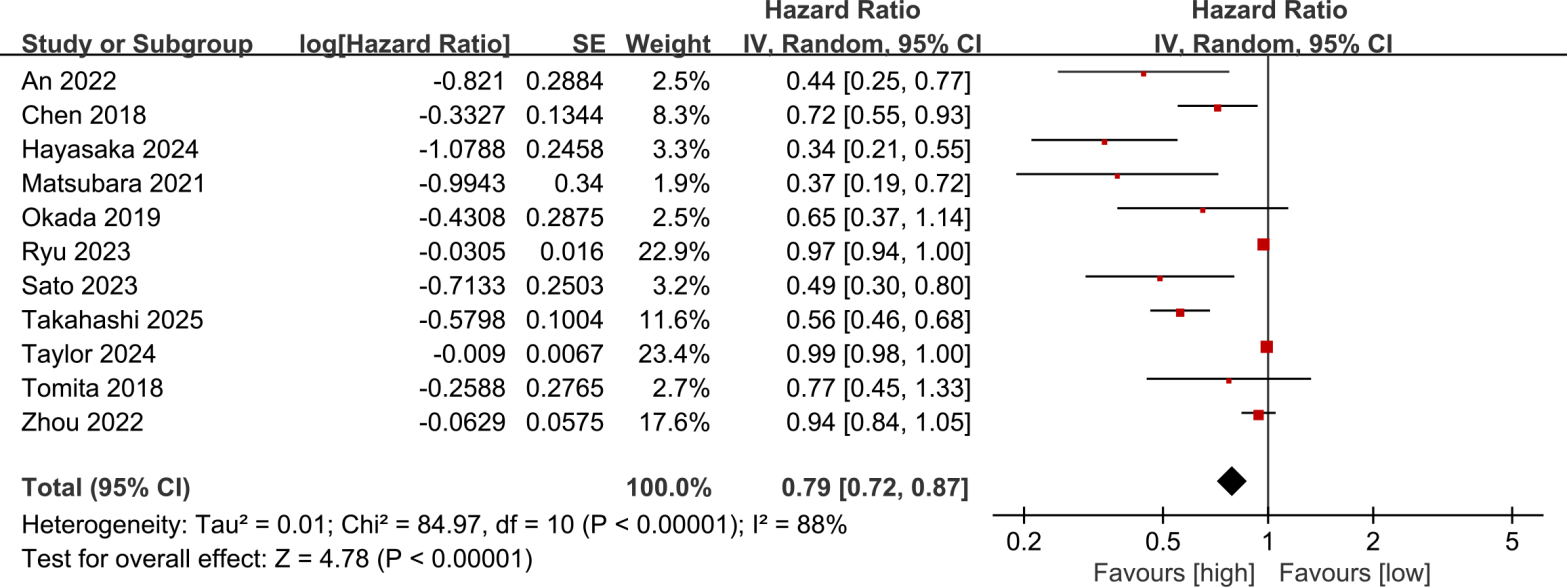
Forest plots of the prognostic value of PNI for OS.

OS results were synthesized from 11 studies. The meta-analysis demonstrated that a higher PNI was significantly associated with longer OS (HR: 0.79; 95% CI: 0.72, 0.87; *P* <0.00001), with substantial heterogeneity (*I*^2^ = 88%, *P* <0.00001) (**Figure 2**). Studies with larger weights had their HR closer to 1, whereas studies with smaller weights had larger fluctuations in effect sizes, which may be a visual manifestation of high heterogeneity (**Figure 2**).

### 3.3 Prognostic value of PNI for RFS

**Figure 3:**
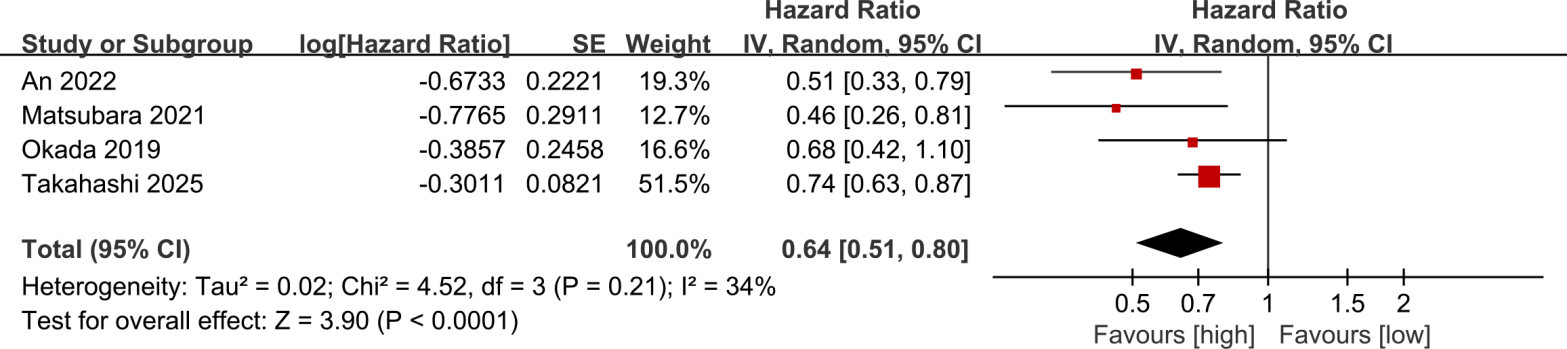
Forest plots of the prognostic value of PNI for RFS.

RFS results were synthesized from 4 studies. The meta-analysis demonstrated that a higher PNI was significantly associated with longer RFS (HR: 0.64; 95% CI: 0.51, 0.80; *P* <0.0001), with no significant heterogeneity (*I*^2^ = 34%, *P* = 0.21) (**Figure 3**).

### 3.4 Prognostic value of PNI for CSS

**Figure 4:**
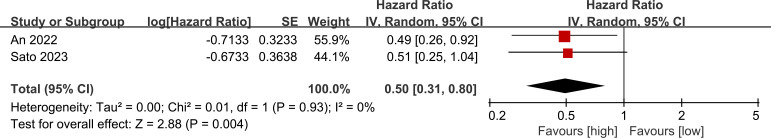
Forest plots of the prognostic value of PNI for CSS.

CSS results were synthesized from 2 studies. The meta-analysis demonstrated that a higher PNI was significantly associated with longer CSS (HR: 0.50; 95% CI: 0.31, 0.80; *P* = 0.004), with no significant heterogeneity (*I*^2^ = 0%, *P* = 0.93) (**Figure 4**).

### 3.5 Publication bias

**Figure 5:**
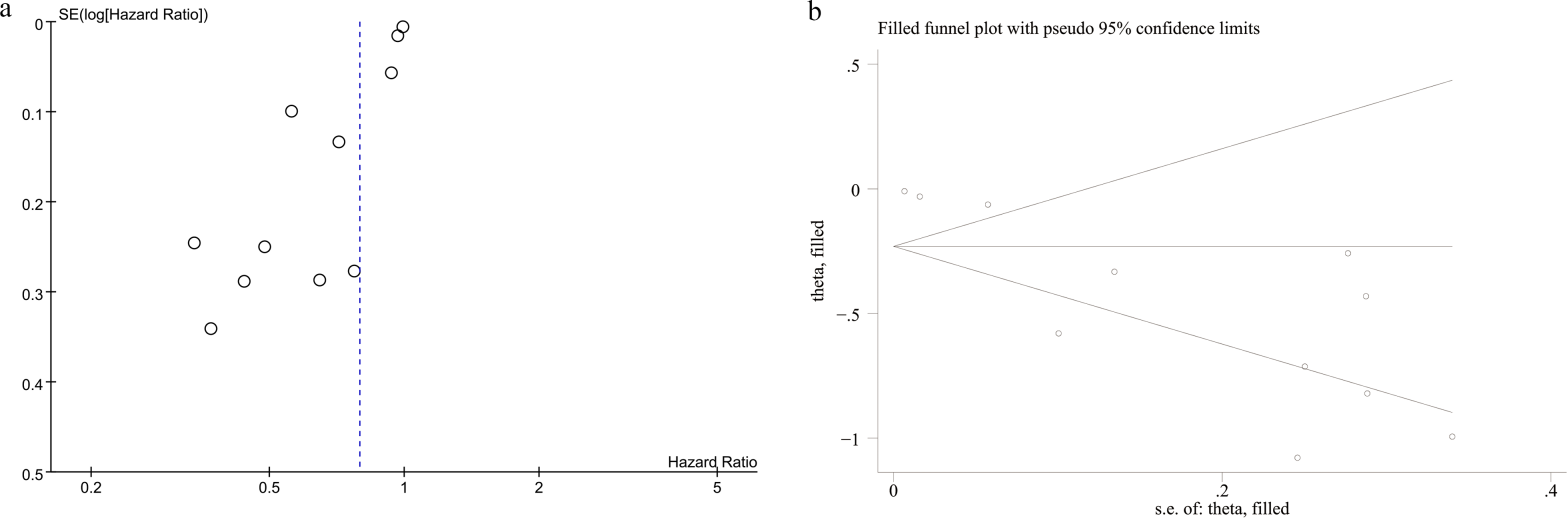
**(A)** Funnel plot of OS and **(B)** Funnel plot of OS after trimming and filling.

The potential publication bias for the prognostic value of PNI for OS was assessed using funnel plots and Egger’s regression test. A significant publication bias was detected for OS (P = 0.0001, **Figure 5A**). The impact of publication bias on OS was assessed using the trim-and-fill method. The results indicated that the correlation between PNI and OS did not change after trimming and filling (HR: 0.79; 95% CI: 0.72, 0.87) (**Figure 5B**).

### 3.6 Sensitivity analysis

**Figure 6:**
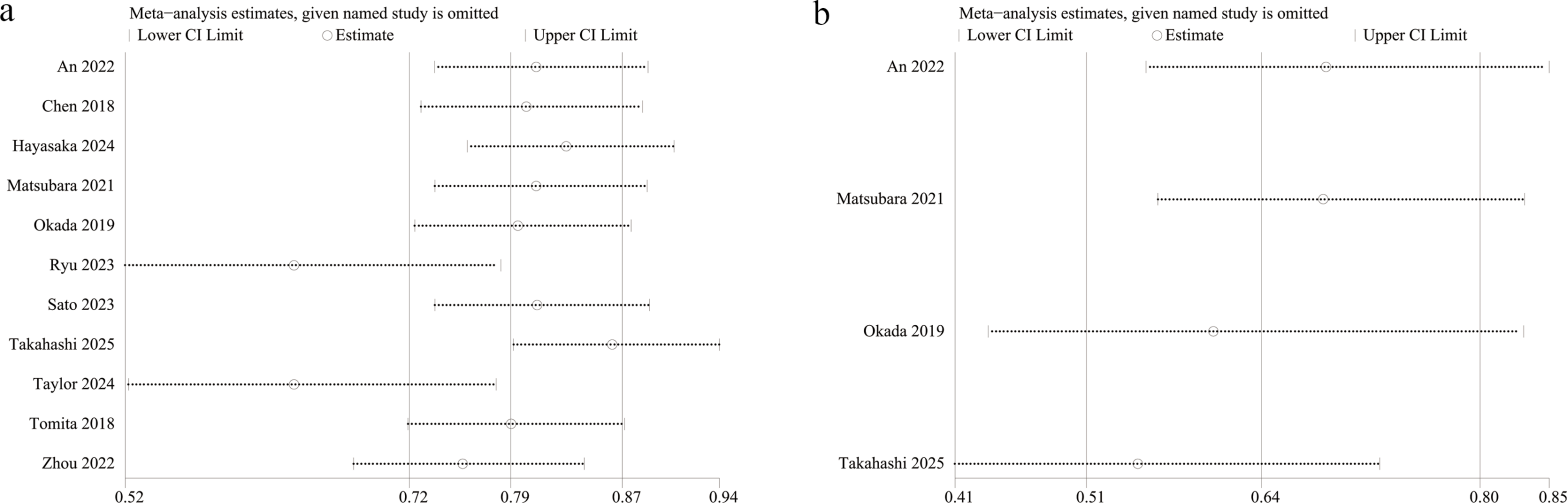
Sensitivity analysis of the prognostic value of PNI for **(A)** OS, **(B)** RFS.

Sensitivity analysis was conducted to evaluate the influence of individual studies on the overall hazard ratios (HRs) for the prognostic significance of PNI for overall survival (OS) and recurrence-free survival (RFS). This was done by sequentially excluding each eligible study. The pooled HRs for both OS (**Figure 6A**) and RFS (**Figure 6B**) remained consistent, indicating the stability and robustness of the results.

## 4 Discussion

This review, through a meta-analysis, systematically evaluated the association of PNI with prognosis in patients undergoing curative surgery for NSCLC for the first time. Results showed that a higher PNI was significantly linked to longer overall survival (HR: 0.79; 95% CI: 0.72, 0.87), RFS (HR: 0.64; 95% CI: 0.51, 0.80), and CSS (HR: 0.50; 95% CI: 0.31, 0.80). These findings were statistically significant (all P < 0.05) and were consistent across different outcome measures. PNI, a simple and cost-effective marker, demonstrates strong prognostic value in surgical patients, likely due to its dual ability to reflect nutritional and immune status (25, 27). Substantial heterogeneity (I² = 88%) was observed in the OS analysis, likely due to differences in the included studies, such as PNI cutoff values, patient populations, and follow-up duration. However, sensitivity analyses demonstrated robust results, with the pooled HRs remaining unchanged after excluding any individual study. Compared with the immunotherapy meta-analysis by Wang et al., the HR values in this study were relatively high, suggesting that the protective effect of PNI in surgical patients may be slightly weaker than in immunotherapy patients (13). However, this reflects the difference in treatment context: immunotherapy directly targets immune pathways, while surgical outcomes are more influenced by nutrition and postoperative recovery. In addition, the heterogeneity of RFS and CSS results in this study was low (I² = 34% and 0%, respectively), indicating that PNI is more consistent in predicting recurrence and cancer-specific death, providing additional support for the clinical application of PNI. However, the lower heterogeneity observed in the RFS and CSS analyses must be interpreted with caution. Due to the limited number of studies included in these analyses, the statistical power of the heterogeneity test is insufficient and may not fully reveal the potential variability between studies. Thus, the conclusions regarding the low heterogeneity of these outcome measures should be considered preliminary and need to be validated with the inclusion of more studies in the future.

In the studies included in this analysis, most of the PNI cut-off values were in the range of 45-50, which may provide empirical reference for future prospective studies to determine a preliminary and clinically operable threshold interval. Identifying a generalizable optimal cutoff is a complex methodologic challenge that may vary according to population characteristics, disease stage, and even geographic region. Future studies should not be limited to reporting a single value, but should use statistical methods such as maximizing the area under the receiver operating characteristic curve or decision curve analysis based on specific clinical outcomes to develop and validate stratified or continuous PNI risk score models adapted to different clinical scenarios in multi-center, large-scale prospective cohorts.

The findings of this study complement those of Wang et al.’s immunotherapy meta-analysis. Wang et al.’s study focused on patients with advanced lung cancer receiving ICIs. The results showed a strong correlation between PNI and survival, and discussed the mechanisms by which PNI affects efficacy through regulation of inflammation and immunotoxicity (13). In contrast, focusing on surgical patients, included 11 studies and covered a wider range of outcomes. Mechanistically, the prognostic value of PNI in surgical patients may be more direct: surgical trauma can lead to decreased albumin and lymphocytopenia, and low PNI may identify patients who are prone to postoperative complications, infection, or delayed recovery, thereby affecting long-term survival (28). In contrast, the role of PNI in immunotherapy may be more related to drug toxicity and immune response. By focusing on surgery, this study expands the scope of application of PNI. Future studies should directly compare the efficacy of PNI in surgery and immunotherapy to optimize individualized treatment strategies.

The biological basis of PNI supports its broad role in cancer prognosis. Serum albumin, a nutritional marker, reflects both protein storage and inflammatory status (29). Low albumin is associated with chronic disease and cachexia, potentially increasing the risk of death by affecting cell repair and immunity (30). Lymphocyte count directly represents immune competence (31). Low lymphocyte count (lymphocytopenia) is common in malnutrition or stress states and may impair anti-tumor immune surveillance (32). In surgical patients, postoperative systemic inflammatory responses can further exacerbate these changes, leading to a decrease in PNI (33). The results of this review are consistent with these mechanisms: patients with higher PNI may have better nutritional reserves and immune function, thereby better tolerating surgical stress and resisting recurrence and metastasis (34).

Heterogeneity and sensitivity analyses in this study provided important insights. The high heterogeneity in the OS analysis (I² = 88%) may be due to the diversity of included studies, including PNI cutoff values, differences in patient characteristics (eg, age, stage), and methodological limitations. The high heterogeneity itself is an important feature of the current evidence and suggests that the prognostic effect of PNI is not a fixed value but is influenced by the clinical setting and measurement methods. However, sensitivity analyses, after excluding individual studies, showed that the pooled HR remained stable, demonstrating that the results were not unduly influenced by individual studies. This strengthens the robustness of our conclusions. In contrast, heterogeneity was lower for RFS and CSS, likely because these outcomes more directly reflect tumor biological behavior and are less affected by confounding factors. Publication bias assessment revealed significant bias in OS (Egger test P = 0.0001), but the results remained unchanged after adjustment for trimming and filling, indicating that bias had a limited impact on the overall conclusions. This bias may have led to a slight overestimation of the effect size of the association of PNI with OS. Although the point estimate is unchanged after the snip-and-fill adjustment, readers should exercise caution in interpreting this HR, which may represent the ‘best estimate’ of the association effect. In the future, more prospective studies with rigorous design and adequate sample size are needed to provide more unbiased evidence. These methodological advantages distinguish this study from the meta-analysis by Wang et al., which did not report bias adjustment in detail. In summary, our analytical framework is more comprehensive and lays a foundation for the application of PNI in surgery.

This study has several limitations. First, all included studies were retrospective in design, which may have introduced selection and confounding bias, although we used the NOS scale to ensure the inclusion of high-quality studies. Second, the study populations were predominantly from Asia, which may limit the generalizability of the results, especially their applicability to Western populations. Third, the cutoff value for PNI was not uniform, which may affect the incorporation of HRs and the establishment of clinical thresholds; future studies should explore the optimal cutoff value. Fourth, short-term outcomes were not included in the analysis. Fifth, missing primary data limited further subgroup analysis. These limitations suggest directions for future research: prospective studies to validate the prognostic value of PNI, the development of standardized cutoff values, and multicenter collaboration to enhance generalizability.

From a clinical perspective, this study suggests that PNI can serve as a simple and cost-effective prognostic tool for patients undergoing NSCLC surgery. Clinicians can assess PNI preoperatively to identify high-risk patients, thereby optimizing perioperative nutritional support, strengthening follow-up, or considering adjuvant therapy. For example, patients with low PNI may benefit from nutritional interventions, which could improve survival outcomes (35). Future studies should explore the relationship between dynamic changes in PNI and prognosis, as well as its combined use with other biomarkers. In addition, the role of PNI in multidisciplinary treatment warrants further investigation, particularly in combination with immunotherapy or targeted therapy. In summary, this systematic review strengthens the prognostic value of PNI in cancer and underscores its unique contribution in the surgical context, offering a new perspective for precision medicine.

## 5 Conclusion

This meta-analysis, based on a systematic review of 11 studies, confirmed that PNI serves as an independent prognostic indicator for patients undergoing radical surgery for NSCLC. A higher PNI was significantly associated with improved OS, RFS, and CSS. Despite limitations such as the retrospective design and variability in PNI cutoff values, sensitivity analyses and trim-and-fill validation confirmed the robustness of the results. It is recommended that PNI be incorporated into preoperative assessments to guide individualized nutritional interventions and postoperative follow-up strategies. Future prospective studies are needed to optimize cutoff values and explore the synergistic effects of dynamic changes in PNI and multidisciplinary treatment.

## Declarations

## Data Availability

The original contributions presented in the study are included in the article/**Supplementary Material**. Further inquiries can be directed to the corresponding author.

## References

[1] Jonna S Al Nusair J Santos GFC Jalal S . Advances in multimodality management of localized non-small cell lung cancer. New York, NY: Thieme Medical Publishers, Inc (2025). 10.1055/a-2715-6723 41043474

[2] Liu X Yang Q Pan L Ye Y Kuang L Xu D . Burden of respiratory tract cancers in China and its provinces, 1990–2021: a systematic analysis of the global burden of disease study 2021. Lancet Regional Health–Western Pacific. (2025) 55. doi: 10.1016/j.lanwpc.2025.101485. PMID: 39968450 PMC11833622

[3] Hu D Yu J Feng J Liu P Chen J-M Zhang H-L . Comparison and trend analysis of cancer incidence in China and globally in 2022. World J Clin Oncol. (2025) 16:107016. doi: 10.5306/wjco.v16.i6.107016. PMID: 40585844 PMC12198859

[4] Tahayneh K Idkedek M Abu Akar F . NSCLC: Current evidence on its pathogenesis, integrated treatment, and future perspectives. J Clin Med. (2025) 14:1025. doi: 10.3390/jcm14031025. PMID: 39941694 PMC11818267

[5] Opalikhin A Friedland S Madariaga ML Owen DH Besse B . Surgical and perioperative advances for patients with locally advanced non–small cell lung cancer. Am Soc Clin Oncol Educ Book. (2025) 45:e481060. doi: 10.1200/edbk-25-481060. PMID: 40505076

[6] Magouliotis DE Cioffi U Minervini F Lampridis S Guttadauro A Scarci M . Changes in quality of life of early-stage lung cancer patients undergoing sublobar resection: a systematic review. Front Surg. (2025) 12:1542036. doi: 10.3389/fsurg.2025.1542036. PMID: 40092396 PMC11906331

[7] Hussein AD Al-Shammari MJ Alqaisy MRK Mohsein OA . Emerging biomarkers in cancer detection and prognosis: a comprehensive review. Int J Immunol. (2025) 7:01–12. doi: 10.33545/26648482.2025.v7.i1a.3

[8] Wu L Tian J-Y Li M-J Jiang F Qiu L-H Yu W-J . Validation of the 9th edition of the TNM staging system for limited-stage small cell lung cancer after resection: a multicenter study. Lung Cancer (Amsterdam Netherlands). (2025) 200:108085. doi: 10.1016/j.lungcan.2025.108085. PMID: 39813875

[9] Takahashi M Tokumasu H Ota S Okada H Aoyama A . Clinical significance of the preoperative prognostic nutritional index on age/comorbidity burdens in patients with resectable non-small cell lung cancer. Surg Today. (2023) 53:681–91. doi: 10.1007/s00595-023-02650-8. PMID: 36720742

[10] Takahashi M Sowa T Tokumasu H Gomyoda T Okada H Ota S . Comparison of three nutritional scoring systems for outcomes after complete resection of non-small cell lung cancer. J Thorac Cardiovasc Surg. (2021) 162:1257–1268.e3. doi: 10.1016/j.jtcvs.2020.06.030. PMID: 32771232

[11] Takahashi M Aoyama A Hamaji M Sozu T Kobayashi M Nakagawa T . Clinical significance of the preoperative prognostic nutritional index in patients with resectable non-small cell lung cancer: a multicenter study. Surg Today. (2025) 55:918–29. doi: 10.1007/s00595-024-02987-8. PMID: 39815110

[12] Taylor M Evison M Michael S Obale E Fritsch NC Abah U . Pre-operative measures of systemic inflammation predict survival after surgery for primary lung cancer. Clin Lung Cancer. (2024) 25:460–467.e7. doi: 10.1016/j.cllc.2024.04.018. PMID: 38796323

[13] Wang L Long X Zhu Y Luo A Yang M . Association of prognostic nutritional index with long-term survival in lung cancer receiving immune checkpoint inhibitors: a meta-analysis. Medicine. (2024) 103:e41087. doi: 10.1097/md.0000000000041087. PMID: 39969311 PMC11688013

[14] Hayasaka K Notsuda H Onodera K Watanabe T Watanabe Y Suzuki T . Prognostic value of perioperative changes in the prognostic nutritional index in patients with surgically resected non-small cell lung cancer. Surg Today. (2024) 54:1031–40. doi: 10.1007/s00595-024-02847-5. PMID: 38700587 PMC11341629

[15] Sato S Sato M Shinohara H . Significance of preoperative evaluation of skeletal muscle index and immune-nutritional status for patients with early-stage non-small cell lung cancer. Gen Thorac Cardiovasc Surg. (2023) 71:354–62. doi: 10.1007/s11748-022-01899-z. PMID: 36562876

[16] He Z Liu K Wu L Wei Q . Nomogram for predicting postoperative cardiopulmonary complications in non-small cell lung cancer based on systemic inflammatory markers: a retrospective study. J Inflammation Res. (2025), 8961–76. doi: 10.2147/jir.s519449. PMID: 40661188 PMC12258541

[17] Page MJ McKenzie JE Bossuyt PM Boutron I Hoffmann TC Mulrow CD . The PRISMA 2020 statement: an updated guideline for reporting systematic reviews. BMJ (Clinical Res Ed). (2021) 372:n71. doi: 10.31222/osf.io/v7gm2. PMID: 33782057 PMC8005924

[18] Wells G Shea B O'Connell D Peterson J Welch V Losos M . The Newcastle-Ottawa Scale (NOS) for assessing the quality of nonrandomised studies in meta-analyses (2011). Available online at: http://www.ohri.ca/programs/clinical_epidemiology/oxford.asp.

[19] Kim SR Kim K Lee SA Kwon SO Lee JK Keum N . Effect of red, processed, and white meat consumption on the risk of gastric cancer: an overall and dose-response meta-analysis. Nutrients. (2019) 11. doi: 10.3390/nu11040826. PMID: 30979076 PMC6520977

[20] Egger M Davey Smith G Schneider M Minder C . Bias in meta-analysis detected by a simple, graphical test. BMJ (Clinical Res Ed). (1997) 315:629–34. doi: 10.1136/bmj.315.7109.629. PMID: 9310563 PMC2127453

[21] An S Han GY Eo W Kim DH Lee S . Comparison of the geriatric nutritional risk index and the prognostic nutritional index in determining survival outcome in patients with non-small cell lung cancer undergoing surgical resection: a cohort study. Medicine. (2022) 101:e31591. 36397370 10.1097/MD.0000000000031591 PMC9666186

[22] Chen Y Wang W Zhang X Yu X Xi K Wen Y . Prognostic significance of combined preoperative platelet-to-lymphocyte ratio and lymphocyte-to-monocyte ratio in patients undergoing surgery with stage IB non-small-cell lung cancer. Cancer Manage Res. (2018) 10:5411–22. doi: 10.2147/cmar.s177320. PMID: 30519089 PMC6234992

[23] Matsubara T Hirai F Yamaguchi M Hamatake M . Immunonutritional indices in non-small-cell lung cancer patients receiving adjuvant platinum-based chemotherapy. Anticancer Res. (2021) 41:5157–63. doi: 10.21873/anticanres.15333. PMID: 34593467

[24] Okada S Shimada J Kato D Tsunezuka H Teramukai S Inoue M . Long-term prognostic impact of severe postoperative complications after lung cancer surgery. Ann Surg Oncol. (2019) 26:230–7. doi: 10.1245/s10434-018-7061-x. PMID: 30456673

[25] Ryu H Song C Kim JS Jeon JH Cho S Kim K . Role of prognostic nutritional index in postoperative radiotherapy for non-small cell lung cancer. Thorac Cancer. (2023) 14:2859–68. doi: 10.1016/j.ijrobp.2022.07.1541. PMID: 37594010 PMC10542465

[26] Tomita M Ayabe T Maeda R Nakamura K . Comparison of inflammation-based prognostic scores in patients undergoing curative resection for non-small cell lung cancer. World J Oncol. (2018) 9:85–90. doi: 10.14740/wjon1097w. PMID: 29988766 PMC6031234

[27] Zhou L Feng F Yang Y Zheng X Yang Y . Prognostic predictors of non-small cell lung cancer treated with curative resection: the role of preoperative CT texture features, clinical features, and laboratory parameters. Clin Radiol. (2022) 77:e765–70. doi: 10.1016/j.crad.2022.06.012. PMID: 35843728

[28] Kıyak R Caglar B . The prognostic nutritional index (PNI) is a powerful biomarker for predicting clinical outcome in gastrointestinal emergency patients: a comprehensive analysis from diagnosis to outcome. Appl Sci. (2025) 15:8269.

[29] Li K Chen Y Zhang Z Wang K Sulayman S Zeng X . Preoperative pan-immuno-inflammatory values and albumin-to-globulin ratio predict the prognosis of stage I–III colorectal cancer. Sci Rep. (2025) 15:11517. doi: 10.1038/s41598-025-96592-5. PMID: 40181140 PMC11968868

[30] Fida S Xu H Weng M Zhou C Ma H Li W . Handgrip strength and platelet-to-albumin ratio as joint prognostic indicator for patients with cancer cachexia. Nutrition. (2025) 136:112794. doi: 10.1016/j.nut.2025.112794. PMID: 40344755

[31] Reichardt L-M Hindelang B Süberkrüb L Hamberger KL Graw JA Schuetze K . Absolute lymphocyte count trajectory predicts clinical outcome in severely injured patients. Eur J Trauma Emergency Surg. (2025) 51:190. doi: 10.1007/s00068-025-02864-0. PMID: 40314767 PMC12048453

[32] Shi J Tang S Shen C Xu D Tian WZ Xu Z . The role of nutritional and inflammatory markers in predicting postoperative complications after esophagectomy for esophageal squamous cell carcinoma: mechanisms, clinical applications, and future perspectives. Front Surg. (2025) 12:1671783. doi: 10.3389/fsurg.2025.1671783. PMID: 41141695 PMC12549710

[33] Wang Y Wang Z Wang H Wang S Bi H Liu J . Correlation analysis of the pindicator of PNI, SII of reoperative immunological and postoperative delirium in patients undergoing thoracoscopic surgery. (2025).

[34] Yun JH Song GJ Son MW Lee MS . Bridging nutrition and oncologic care: addressing malnutrition in adjuvant chemotherapy for gastric cancer. Foregut Surg. (2025) 5:17–30. doi: 10.51666/fs.2025.5.e2

[35] Kaya SO Akcam TI Ceylan KC Samancılar O Ozturk O Usluer O . Is preoperative protein-rich nutrition effective on postoperative outcome in non-small cell lung cancer surgery? A prospective randomized study. J Cardiothoracic Surg. (2016) 11:14. doi: 10.1186/s13019-016-0407-1. PMID: 26782276 PMC4717613

