# Supplementary material for: Association between the prognostic nutritional index and prognosis in patients with non-small cell lung cancer undergoing curative lung resection: a systematic review and meta-analysis
Source: Front Oncol. 2026 May 21;16:1834210. doi: 10.3389/fonc.2026.1834210 (PMC13233355; doi:10.3389/fonc.2026.1834210)
Supplement: Supplementary file 1 [file Table1.docx]

TableS1 Literature search strategy

Pubmed-156

(("Carcinoma, Non-Small-Cell Lung"[Mesh]) OR (((((((Non-Small-Cell Lung Carcinomas) OR (Non-Small Cell Lung Cancer)) OR (Non-Small-Cell Lung Carcinoma)) OR (Non Small Cell Lung Carcinoma)) OR (Nonsmall Cell Lung Cancer)) OR (Non-Small Cell Lung Carcinoma)) OR (NSCLC))) AND ((prognostic nutritional index[Title/Abstract]) OR (PNI))

Embase-251

((Carcinoma, Non-Small-Cell Lung or (Non-Small-Cell Lung Carcinomas or Non-Small Cell Lung Cancer or Non-Small-Cell Lung Carcinoma or Non Small Cell Lung Carcinoma or Nonsmall Cell Lung Cancer or Non-Small Cell Lung Carcinoma or NSCLC)) and (prognostic nutritional index or PNI)).af.

Cochrane-7

((Carcinoma, Non-Small-Cell Lung or (Non-Small-Cell Lung Carcinomas or Non-Small Cell Lung Cancer or Non-Small-Cell Lung Carcinoma or Non Small Cell Lung Carcinoma or Nonsmall Cell Lung Cancer or Non-Small Cell Lung Carcinoma or NSCLC)) and (prognostic nutritional index or PNI)).af.

WOS-263

((Carcinoma, Non-Small-Cell Lung) OR (((((((Non-Small-Cell Lung Carcinomas) OR (Non-Small Cell Lung Cancer)) OR (Non-Small-Cell Lung Carcinoma)) OR (Non Small Cell Lung Carcinoma)) OR (Nonsmall Cell Lung Cancer)) OR (Non-Small Cell Lung Carcinoma)) OR (NSCLC))) AND ((prognostic nutritional index) OR (PNI)) (Topic)
